# Nutritional Optimization of the Surgical Patient: A Narrative Review

**DOI:** 10.1016/j.advnut.2024.100351

**Published:** 2024-11-29

**Authors:** Olivia Heutlinger, Nischal Acharya, Amanda Tedesco, Ashish Ramesh, Brian Smith, Ninh T Nguyen, Paul E Wischmeyer

**Affiliations:** 1 School of Medicine, University of California–Irvine, Irvine, California, United States; 2 Department of Surgery, University of California–Irvine, Irvine, California, United States; 3 Department of Anesthesiology, Duke University School of Medicine, Durham, North Carolina, United States; 4 Department of Surgery, Duke University School of Medicine, Durham, North Carolina, United States

**Keywords:** nutritional optimization, surgical nutrition, nutritional screening tools, perioperative nutrition guidelines, perioperative nutrition screening

## Abstract

An increasing body of literature supports the clinical benefit of nutritional assessment and optimization in surgical patients; however, this data has yet to be consolidated in a practical fashion for use by surgeons. In this narrative review, we concisely aggregate emerging data to highlight the role of nutritional optimization as a promising, practical perioperative intervention to reduce complications and improve outcomes in surgical patients. This review of the surgical nutrition literature was conducted via large database review. There were no distinct inclusion/exclusion criteria for this review; however, we focused on adult populations using up-to-date literature from high-quality systematic reviews or randomized controlled trials when available. Current perioperative management focuses on the mitigation of intraoperative and immediate postoperative complications. Well-defined risk calculators attempt to stratify patient surgical risk preoperatively to reduce adverse events directly related to surgical procedures, such as hemorrhage, cardiopulmonary compromise, or infection. However, there is a lack of standardization of prognostic tools, nutritional protocols, and guidelines governing the assessment, composition, and administration of nutritional supplementation. Substantial data exist demonstrating the clinical benefit in the operative setting. In this work, we provide a fundamental primer for surgeons to understand the clinical importance of nutritional optimization along with practical prognostic tools and recommendations for use in their practice. While the extent to which nutritional optimization improves patient outcomes is debatable, the evidence clearly demonstrates a clinically meaningful benefit. Evaluating nutritional status differs based on disease severity and etiology of presentation, thus surgeons must select the appropriate prognostic tools to assess their patients during the perioperative period. This information will catalyze subsequent work with a multidisciplinary team to provide personalized dietary plans for patients and spark research to establish protocols for specific presentations.

**Statement of Significance**

This review provides an organized, centralized reference where surgeons can review the complex topic of perioperative nutrition and access both key screening tools and recommendations based on their patient’s presentation.

## Introduction

Over the past decade, surgeons have sought to identify factors outside of the operating room that may improve surgical outcomes. The development of Enhanced Recovery After Surgery protocols have greatly enhanced the quality and speed of patient recovery, and there is robust data that supports improved long-term survival rates and reduced complications, hospital length of stay (LOS), readmission rates, and hospital costs with protocol compliance [[1], [2], [3]]. Despite these findings, questions remain about the intricate relationship between clinical outcomes and nutritional deficiency. The postoperative period represents a critical phase of surgical care and is characterized by a surge in metabolic demand, increased oxidative stress and inflammation, heightened immune activity, and a delicate balance of protein synthesis and catabolism [4]. This state, while essential for tissue healing, can be tipped out of balance by poor nutrition and contribute to suboptimal surgical outcomes. Preoperative nutritional supplementation, continued intake with carbohydrate-dense clear liquids ≤2 h before surgery, and immediate resumption of oral intake after surgery have all been demonstrated to decrease infection risk, hospital LOS, mortality, and more [[5], [6], [7], [8], [9], [10], [11], [12], [13], [14], [15], [16]].

Surgeons, as the vanguards of surgical care, play an essential role in identifying patients who may benefit from perioperative nutritional support. A nationwide survey sent to gastrointestinal (GI) and oncologic surgical faculty showed that 74% of surgical faculty agreed that malnutrition is a major problem, 83% acknowledge that preoperative nutritional supplementation reduces complications, and 81% acknowledge that a standard protocol for nutritional optimization would be beneficial [17]. However, only 21% of patients were reported to receive preoperative nutritional supplementation, and 22% were supplemented postoperatively [17]. While consideration of nutritional status is well-known to surgeons and surgical trainees, practical information on how to evaluate and optimize nutrition is lacking in surgical education and training. This is of particular importance when considering the breadth of complexity of general surgery patients, where the nutritional needs of a healthy patient undergoing a mild surgery are vastly different than a comorbid or oncologic patient undergoing a lengthy and complex surgery. As such, addressing this gap is of paramount importance to enhance patient care and surgical outcomes.

## Methods

We conducted a narrative review of the literature on surgical nutrition. Our search was conducted via PubMed, Google Scholar, and ScienceDirect primarily using the search terms “surgical nutrition,” “perioperative nutritional optimization,” and “nutritional status screening tools AND surgery.” We did not include any literature outside of traditional publishing and distribution channels. These search terms were used to conduct a comprehensive review on this topic, after which more specific search terms were used to highlight special circumstances where nutrition is particularly important, that is, open abdomens, burn injuries, critical care, bariatric surgery, and cytoreductive surgery (CRS).

There were no distinct inclusion/exclusion criteria for this review, and we report no biases in this work. No definitive date range was established for manuscripts included in our review; however, we intentionally selected the most updated sources when able. When available, systematic reviews and meta-analyses were chosen for review, followed by multicenter randomized control trials (RCT)s, single-center RCTs, and retrospective studies with the aim of providing a comprehensive review of the current literature and understand trends within the field of surgical nutrition in adults (age older than 18 y).

## Discussion

### Evaluating nutritional status

#### Nutritional risk screening tools

Identifying patients who are malnourished or at risk of malnutrition continues to be a challenge in the elective setting. The European Society for Clinical Nutrition and Metabolism (ESPEN) defines malnutrition in patients meeting 1 of the following 2 criteria: *1*) BMI (in kg/m^2^) of <18.5 or *2*) the combined finding of unintentional weight loss and either reduced BMI or a low fat-free mass index [18]. Unintentional weight loss includes weight loss of >10% over an indefinite time or >5% over 3 mo. Reduced BMI is <20 or <22 for patients younger or older than 70 y, respectively. The most widely used nutrition risk screening tools for hospitalized patients or those undergoing elective surgery are the Nutritional Risk Screening (NRS)-2002, Subjective Global Assessment (SGA), Nutrition Risk Index, and Perioperative Nutrition Score (Table 1) [[19], [20], [21], [22], [23]]. The NRS-2002 score has been validated for elective surgical and critically ill patients. This scoring system is recommended by ESPEN and can predict the risk of postoperative complications and prolonged hospital LOS [16,[24], [25], [26]]. A recent meta-analysis determined the NRS-2002 to be independently associated with increased risk of postoperative complications and worse overall survival in patients with cancer, suggesting its clinical utility in this population [27]. The Nutrition Risk Index was developed specifically for cancer patients in mind, being both sensitive and specific for identifying patients at risk of developing surgical complications [28]. The SGA incorporates both patient history and physical examination and has demonstrated similar efficacy to the NRS-2002 score in geriatric patients. The Perioperative Nutrition Score was developed specifically for surgical malnutrition risk [29] and has been validated to predict risk of adverse postoperative outcomes, even independent of a validated malnutrition diagnosis [23]. Thus, each of these screening scores have data recommending their use, and none have been demonstrated to be superior to one another. Any combination of these assessment tools may be used to reflect a more accurate picture of nutritional status. The selection can be tailored to the individual’s disease history [[19], [20], [21], [22], [23]].

**TABLE 1:** Comparison of nutritional screening tools.

	NRS-2002 [19]	mNUTRIC [20]	SGA [21]	NRI [22]	PONS [23]
Contributing variables	Prescreening: BMI <20.5 kg/m^2^ Weight loss within 3 mo Reduced dietary intake in the last week ICU status Screening (if yes to any one of the above): Nutritional impairment Severity of disease Age ≥ or <70 y	Age Number of comorbidities Days from hospital to ICU admission APACHE II score SOFA score Not included in mNUTRIC: IL-6	Weight change Dietary intake GI symptoms lasting ≥2 wk Functional capacity Disease and its relation to nutritional requirements Included in the 7-point SGA: Loss of subcutaneous fat Signs of muscle wasting	Serum albumin Present weight Usual weight (stable weight ≥6 mo before surgery) [1.519 × serum albumin level (g/L)] + [0.417 × current weight/usual weight)] × 100	BMI <18.5 (<20 if older than age 65 y) Weight loss > 10 lbs in last 3 mo without trying Eating less than half normal meals in last week Albumin < 3.5 (g/L)
Scoring system	0–7 ≥3: patient at nutritional risk <3: repeat screening weekly	0–9 ≥5: patient at high risk of malnutrition <5: patient at low risk of malnutrition	7–35 7: normal 8–14: mild to moderate malnutrition 15–35: severe malnutrition	>100: no malnutrition 97.5–100: mild malnutrition 83.5–97.4: moderate malnutrition <83.5: severe malnutrition	0–4: Any positive answer recommends referral to a registered dietitian-nutrition clinic or need for perioperative nutrition therapy
ESPEN endorsed	Yes	No	Yes	No	No
ASPEN endorsed	Yes	Yes	Yes	No	No
Validated patient population	ICU patients Elective surgery	ICU patients Burn patients	Hospitalized patients General population Cancer patients	Cancer patients	Elective surgery

Abbreviations: APACHE II, Acute Physiology and Chronic Health Evaluation II; ASPEN, American Society for Parenteral and Enteral Nutrition; ESPEN, European Society for Parenteral and Enteral Nutrition; GI, gastrointestinal; ICU, intensive care unit; mNUTRIC, modified Nutrition Risk in Critically Ill; NRI, Nutrition Risk Index; NRS, Nutritional Risk Screening; PONS, Perioperative Nutrition Score; SGA, Subjective Global Assessment; SOFA, Sequential Organ Failure Assessment.

In January 2016, the Global Leadership Initiative on Malnutrition developed a 2-step approach for diagnosing malnutrition [30]. The first step is the identification of at-risk patients using any validated screening tool, and the second step involves assessment and grading of malnutrition severity via 3 phenotypic criteria (unintentional weight loss, low BMI, and reduced muscle mass) and 2 etiologic criteria (reduced food intake and inflammation/disease burden) [30]. A systematic review evaluating the ability of the Global Leadership Initiative on Malnutrition criteria to identify malnutrition in the hospital setting found that many studies validated this tool as both sensitive and specific for identifying inpatient malnutrition with good agreement between screening and nutritional assessment methods [31]. While further data are needed to validate this tool, it may be beneficial in assessing nutritional status by combining subjective and objective patient factors.

#### Nutritional biomarkers

To fully evaluate preoperative status and monitor postoperative progression, adjuvant analysis of objective nutritional markers is necessary. Albumin and prealbumin are considered traditional serum markers of nutritional status, with prealbumin preferred for its shorter half-life that reflects acute changes in nutritional status [32]. However, more recent data have rejected the utility of both laboratory values as definers of malnutrition and favor them to be markers associated with nutrition risk [33]. Serum albumin, absolute lymphocyte count, and the SGA have been shown to accurately predict postoperative complications after abdominal surgery [34]. It is important to note that markers such as BMI, midarm circumference, and tissue skinfold thickness may not correlate with postoperative complication risk [35], bolstering the notion that malnutrition may not be obvious based on appearance. Although this finding contrasts with ESPEN’s BMI-based definitions for malnutrition, it may be more applicable to BMI ranges falling outside of the ESPEN defined parameters. This complexity further supports the need for other more comprehensive, yet practical, prognostic tools that can be leveraged by clinicians.

#### Procedural considerations

In the elective setting, the evaluation of nutritional status depends on the extent and radicality of surgery being performed and the patient’s baseline health. A large prospective study was conducted to evaluate the predictiveness of the SGA and NRS-2002 for general surgery patients divided into 3 groups based on the complexity of the surgery being performed [35]. Group 1 included major surgical operations for GI malignancies (pancreas, liver), group 2 included moderate surgical patients (cholecystectomy, hernia repair), and group 3 included minor surgical patients (hemorrhoidectomy, pilonidal cyst excision) [35]. Both SGA and NRS-2002 showed significant correlation with postoperative complications for all groups, LOS for groups 1 and 2, and mortality for group 1 [35], highlighting the importance of evaluating nutritional status in all surgical patients regardless of surgical complexity. Nutritional deficiency can be particularly difficult to assess in trauma, burn, and critically ill patients, where massive resuscitation invalidates weight and BMI as markers of nutrition status and albumin and prealbumin will decrease as negative acute phase reactants, thus are unreliable in this setting. The two best evidence-based prognostic tools in these patient populations are the NRS-2002 and the Modified Nutrition Risk in Critically Ill (mNUTRIC) systems (Table 1). The NRS-2002 has the strongest predictive value per ESPEN guidelines, and both the Society of Critical Care Medicine and American Society for Parenteral and Enteral Nutrition (ASPEN) recommend its use for patients in the intensive care unit (ICU) [36]. The mNUTRIC score has been validated by several studies to correlate with clinical outcomes, serve as an independent predictor of mortality in the surgical ICU, and be independently associated with 28-day mortality [[37], [38], [39], [40]]. While the Society of Critical Care Medicine and ASPEN endorse this scoring system, ESPEN does not recommend mNUTRIC’s use as its incorporation of prehospital data may not account for the acute inpatient nutritional decompensation that is the main concern in these patients [20]. For patients with >30% total body surface area burns, mNUTRIC has been shown to be superior to NRS-2002 in predicting 28-day mortality [41]. In sum, both the NRS-2002 and mNUTRIC scores can be used to accurately predict hospital mortality, although caution should be taken when using mNUTRIC scores for patients with prolonged hospital LOS [20,42]. In the setting of burn injuries, mNUTRIC may be superior for nutritional evaluation.

#### Special patient populations—cancer

There are additional parameters that must be considered in high-risk patients, particularly those with neoplastic disease. Both NRS-2002 and SGA have been validated for patients undergoing major abdominal surgery [[43], [44], [45]], with the NRS-2002 being more specific and the SGA more sensitive for patients with cancer [43]. Low preoperative prealbumin, albumin, total lymphocyte count, BMI, and anorexia have been demonstrated to be independent predictors of postoperative complications [[46], [47], [48], [49]], and these factors may be useful in elucidating patients who will benefit the most from perioperative nutritional optimization. A prospective RCT evaluated the role of preoperative nutritional supplementation for patients with cancer without signs of clinical malnutrition and found significantly lower incidence of serious complications and improved serum nutrition markers (albumin, total protein, transferrin, and total lymphocyte count) in the interventional group [50]. While the degree of benefit of nutritional optimization may vary with the complexity of the patient or surgery, the literature supports improvement in many patient types, and thus, screening and consideration of these factors is of utmost importance.

#### Special patient populations—obesity

Patients classified as obese represent a particularly difficult population to assess for nutritional status, as many patients experience nutrient deficiencies despite the lack of undernourishment. The so-called obesogenic diet often contains a large portion of processed foods rich in carbohydrates, saturated fats, and sodium with deficiencies in protein and micronutrients including vitamins [A, thiamine (vitamin B-1), folate (vitamin B-9), cobalamin (vitamin B-12), C, D, and E], iron, zinc, magnesium, and calcium [[51], [52], [53]]. These patients are also at very high risk of long-term nutrient deficiencies should they undergo bariatric surgery. Nutritional risk screening tools have limited use in this population as many incorporate body weight as an important factor in their assessment.

### Elective general surgery

Nutritional optimization in the perioperative period plays an important role in surgical outcomes. Approximately 40%–50% of patients who undergo surgery have some degree of malnutrition, a percentage that is often underestimated [54,55]. Many factors increase risk for malnutrition, including chronic inflammatory states, poor oral intake secondary to pain and dysphagia, malabsorptive syndromes, and malignancy [21]. Additionally, all patients are susceptible to weight loss during hospitalization, occurring in up to two thirds of patients [55]. Poor nutritional status is a known independent risk factor for adverse outcomes across general surgery subspecialties, including increased hospital LOS, readmission rates, rates of infection, hospital costs, and 30-d mortality [54,56,57]. While the nutritional status of patients who are obviously malnourished is typically addressed, the optimization of patients who are adequately nourished or mild to moderately malnourished is generally neglected in the setting of elective surgery.

#### Preoperative nutrition

Transient insulin resistance can last from a few days to 3 wk after an uncomplicated, elective abdominal surgery [15]. This may be exacerbated by a prolonged preoperative fasting state. Early studies have demonstrated that administration of a glucose infusion the night before abdominal surgery decreases protein catabolism, insulin resistance, and fat oxidation, improving postoperative metabolism by attenuating the release of cortisol and glucagon [[58], [59], [60]]. Preoperative carbohydrate solutions are known to reduce insulin resistance by ≤50%, prevent muscle breakdown, decrease hospital LOS, reduce time to recovery, and improve postoperative function while simultaneously decreasing patient-reported preoperative thirst, hunger, and anxiety [[61], [62], [63], [64], [65]]. The typical formulation uses maltodextrin carbohydrate polymers to reduce solution osmolality. As little as 400 mL of this solution is sufficient to stimulate a hormonal change from the fasted to fed state without delaying gastric emptying or increasing the risk of adverse effects [62]. These findings are consistent among patients undergoing both major and minor abdominal surgeries [66]. Several high-quality articles have confirmed that oral administration of carbohydrate-containing clear liquids reduces postoperative insulin resistance and risk of infection, and their administration ≤2 h before surgery is overwhelmingly supported in the literature [[5], [6], [7]].

Aside from perioperative carbohydrate supplementation, significant efforts to elucidate the role of protein, fat, and specific amino acids in mitigating postsurgical whole-body catabolism are ongoing. The role of “prehabilitation,” that is, supplementation of carbohydrate and protein containing or immune-enhancing nutrition in the preoperative period, has been studied to evaluate its effect on surgical outcomes. While some studies have identified mild evidence supporting its benefit on insulin resistance and hospital LOS [14,15], others have been inconclusive on whether prehabilitation has any effect on postoperative outcomes [67]. Current guidelines recommend the use of preoperative parenteral nutrition (PN) in severely malnourished patients only, as its associated with significantly reduced postoperative complication rates in those who are unable to adequately nourish via oral or enteral intake [13,68,69]. Providing preoperative nutritional support to abdominal surgical patients with an NRS-2002 score of ≥5 is associated with decreased complication rates and shorter hospital LOS [70]. As such, this clinical tool can be used to identify patients most likely to benefit from preoperative nutritional optimization.

#### Postoperative nutrition

In most cases, oral nutrition should be initiated immediately after surgery to reduce overall complications and shorten hospital LOS [21]. Oral feeding has been shown to be safe in patients with new lower GI anastomoses and may reduce the duration of ileus, shorten hospital LOS, and reduce mortality [[8], [9], [10], [11], [12]]. A 2023 meta-analysis on early oral feeding for upper GI surgeries, including those for esophageal and gastric tumors, reported earlier return of bowel function and shorter hospital LOS with no increase in complications [71]. When oral nutrition is not feasible, enteral nutrition (EN) should be attempted first. If unsuccessful within 72 h in reaching goal, then PN or supplemental parenteral nutrition (SPN) should be started. The literature regarding the use, safety, and benefit of early PN has changed significantly in the last few years. Recent data in 4 large RCTs of acutely ill patients, including surgical patients, has shown that PN no longer is associated with any increased risk of infection of any kind [[72], [73], [74], [75], [76]]. In fact, the new ASPEN guidelines [72] highlight that EN and PN may be used interchangeably and, when goal EN is not feasible, that early PN is safe and effective and results in similar outcomes to EN. Finally, in support of early PN use in surgical patients, a recent trial in major abdominal surgical patients demonstrated that initiating SPN at day 3 significantly reduced infectious complications compared with waiting until day 8 for SPN [77].

#### Immunonutrition

There is significant debate over supplementation with so-called immune-modulating agents, nutritional factors [i.e. glutamine, arginine, and ω-3 (n–3) fatty acids] thought to play a unique role in supporting the immune system that could pose a benefit in overall recovery and infection prevention when supplemented in the acute period after elective surgery. A systematic review of glutamine supplementation in patients after major elective abdominal surgery showed a statistically significant reduction in hospital LOS (weighted mean difference: −2.67; *P* < 0.0001) with no effect on overall morbidity [78]. However, the included RCTs were underpowered, and other RCTS and meta-analyses on glutamine have yielded inconclusive results, so more data are needed to determine its utility [[78], [79], [80]]. A key meta-analysis of arginine-supplemented or immunonutrition diets in surgery found a significant association with reduced perioperative infectious complications, decreased risk of anastomotic leakage, and reduced hospital LOS [81]. The benefit was similar whether arginine supplementation was given preoperatively only, preoperatively and postoperatively, or only postoperatively. A systematic review of 35 RCTs on arginine-supplemented diets in patients undergoing both GI and non-GI elective general surgery found significant reductions in overall infectious complications (risk ratio: 0.59, *P* < 0.00001) and hospital LOS (weighted mean difference: −2.38; *P* < 0.00001) [82]. A 264-patient RCT demonstrated a reduction in infectious complications (23.8% compared with 10.7%; *P* = 0.0007) and wound infections (16.4% compared with 5.7%; *P* = 0.0008) with the use of an arginine-supplemented nutrition drink when compared with that in the use a standard high-calorie supplement [83]. The role of immunonutrition has been most widely studied in patients with upper GI cancers, with the most recognized benefits being reductions in infectious complications and hospital LOS [[84], [85], [86]]. The ESPEN guidelines formally recommend perioperative or postoperative immunonutrition for malnourished patients undergoing major cancer surgery, but further data are needed to give recommendations on its role in noncancer surgery [16,85,86]. The role of perioperative prebiotics, probiotics, and symbiotics in reducing infectious complications is currently under investigation, although more data are needed to guide formal recommendations [87].

### Metastatic cancer and CRS

Patients with metastatic disease undergoing major CRS are a particularly high-risk group that must be considered. A literature review of preoperative, modifiable factors that may influence postoperative complications in patients undergoing CRS and hyperthermic intraperitoneal chemotherapy found poor nutritional status and sarcopenia to be significantly associated with postoperative complications, risk of infection, hospital LOS, mortality, and overall survival [88]. Several studies found that implementation of multimodal prehabilitation in patients undergoing CRS for advanced ovarian cancer is associated with decreased hospital LOS, rate of readmission, and intraoperative complications [[89], [90], [91]]. Following discussions at the 10th Peritoneal Surface Malignancy Workshop, a group of surgical oncologists provided recommendations for optimization of patients undergoing CRS and hyperthermic intraperitoneal chemotherapy, including provision of ≥1.2–2.0 g/kg protein equivalents/d, preoperative and postoperative immunomodulatory supplementation, and PN in the immediate postoperative period until oral intake can cover daily requirements [92]. These recommendations are an important starting point when considering this patient population; however, more RCTs are needed to guide further recommendations.

#### Bariatric surgery

Patients undergoing bariatric surgery frequently follow strict preoperative and postoperative diets that vary depending on the type of bariatric surgery done. Preoperative low-calorie diet and ω-3 supplements have been shown to decrease liver volume and visceral fat as well as improved glycemic control, wound healing, and lower surgical complications depending on the specific diet type followed [93]. Bariatric patients proceed through a 3-step diet advancing from clear liquids to full liquids to puree/soft diet before moving onto solid foods [93]. Surgical techniques that focus on restriction, including sleeve gastrectomy and gastric banding, can affect absorption of iron, selenium, and vitamin B-12 [94]. Anatomy-altering surgeries, most commonly Roux-en-Y gastric bypass, lead to much more pronounced vitamin and nutrient deficiencies, including vitamins A, C, D, B-1, B-2, B-6, and B-12, zinc, and copper [94,95]. Multidisciplinary evaluation intervention among a team of surgeons, dietitians, and medical doctors for management of comorbidities is imperative for long-term follow-up in these patients.

### Trauma and critical care

Critical illness and traumatic injuries pose a unique challenge for surgeons in terms of nutrition, as these patients’ baseline nutritional status is often unknown and ongoing nutritional needs vary over their postinjury course. Many of these patients require serial procedures and/or surgeries that cause subsequent insults and lead to interruptions in nutrient delivery, adding to the complexity of this population. After a traumatic injury, tissue damage triggers an acute phase response characterized by protein catabolism, insulin resistance, decreased glucose uptake by cells, sodium and water retention, and increased lipid metabolism [96]. The physiologic insult caused by trauma is significant, and energy expenditure has been shown to be 20%–50% greater in trauma patients compared with those undergoing elective surgery [96,97]. This prompt nutritional deterioration leads to diminished immunoreactivity and increased susceptibility to infection [96]. As such, malnutrition is associated with higher morbidity and mortality, delayed wound healing, delayed mobilization, prolonged hospital and ICU LOS, as well as higher rates of reoperation and readmission [96].

Caloric needs increase by as much as 1.7-fold in trauma patients, a number that may be further exasperated by newer early mobility protocols [98]. While EN is preferred as the initial nutrition delivery route over PN when available [20,99,100], the specific composition of the enteral feeds is seldom considered. Achieving target protein intake is known to decrease mortality rates in critically ill patients [101,102]. The ASPEN/ESPEN guidelines recommend protein intake on the order of 1.2–2.0 g/kg/d, although large-scale surveys have shown that patients in the ICU receive an average of only 0.6 g/kg/d [20,21,98]. More recently, the EFFORT Protein Trial found that in mechanically ventilated, critically ill patients, higher protein doses did not improve time to discharge and potentially worsened outcomes for those with acute kidney injuries and high organ failure scores [103]. The NUTRIREA-3 trial compared low and standard protein and calorie targets (6 kcal/kg/d and 0.2–0.4 g/kg/d protein compared with 25 kcal/kg/d and 1.0–1.3 g/kg/d protein) in mechanically ventilated patients requiring vasopressors and found no significant difference in death, time to ICU discharge, and infection rates between the 2 groups [104]. The low target group, however, had lower rates of emesis, diarrhea, bowel ischemia, and liver dysfunction, suggesting lower protein supplementation may confer greater outcomes [104]. Protein and calories should be slowly titrated up over first 3–5 days to optimize outcomes [105]. Finally, recent data indicate that calorie or energy targets should be personalized via use of indirect calorimetry [105]. Predictive equations have been shown to be highly inaccurate in predicting calorie needs, with correlation factors of 0.24–0.73 for 12 equations tested [106,107]. The use of indirect calorimetry to guide energy targets has been associated with a reduction in ICU-related mortality in systematic reviews, and both ESPEN and ASPEN guidelines advocate for indirect calorimetry to measure energy/calorie targets [20,[108], [109], [110], [111]].

### Nutrition and the open abdomen

Protein, fluid, and electrolyte loss can lead to detrimental outcomes in patients with surgically open abdomens. Nutritional support in this patient population tends to be suboptimal due to interruptions for reoperation and transport, patient intolerance, delivery-related complications, and fear of morbidity-increasing complications (gastric retention, bowel edema, bloating, vomiting, aspiration, and diarrhea) [99,112]. However, evidence shows that early EN is associated with decreased incidence of ventilator-associated pneumonia (43.8% compared with 72.1%; *P* = 0.008), lower rates of fistula formation (9% compared with 26%; *P* = 0.05), and decreased mortality (11.1% for EN within 14 d compared with 47.8% for EN after 14 d; *P* < 0.001) [[113], [114], [115]]. The protein requirement for these patients increases ≤2.5 g/kg/d, as ∼2–4.6 g of nitrogen are lost per liter of abdominal fluid output depending on the type of temporary abdominal closure used [112,116]. Early EN is beneficial in patients with a viable GI tract (bowel length of >75 cm) [117] and early PN should be considered for patients in bowel discontinuity or with prolonged ileus, as data show it reduces infectious complications and poses no greater risk than EN [[72], [73], [74], [75], [76], [77]]. We are currently engaged in a U.S. Department of Defense-funded RCT studying the role of indirect-calorimetry guided nutrition throughout hospital stay in patients with traumatic abdominal injury who commonly have open abdomens. This study uses early PN started within 72 h in the experimental group guided by indirect calorimetry twice weekly in the ICU and examines physical function, as measured by the 6-min walk test, as the primary end point (SEND Home Study- ClinicalTrials.gov Identifier: NCT06065202).

### Burn injuries

Acute thermal injuries are known to cause a profound hypermetabolic response that can persist for up to 2 y postinjury in severe cases [118]. Resting metabolic rate has been shown to reach up to 180% of basal rate in the first week following thermal injury and remains elevated to 130%–150% at the time of full healing, 120%–140% at 6 mo, and 110%–120% at 12 mo per the Harris–Benedict equation [118,119]. Early EN (within 24 hours) has been shown to decrease muscle protein catabolism, improve wound healing, decrease ICU and hospital LOS, diminish rates of sepsis, decrease infectious load, and decrease the risk of Curling ulcer formation [118,120,121]. Evaluation of the evolving metabolic needs in these patients is imperative, with serial indirect calorimetry regarded as the best option to fully meet energy targets. At institutions where indirect calorimetry is not available, the Toronto formula has been shown to be the most accurate proxy of energy expenditure. These tools can provide invaluable data when caring for burn patients, as the lack of specific guidelines or protocols for burn resuscitation poses a great challenge to the surgical team.

Excess carbohydrate provision after thermal injuries can propagate hyperglycemia with subsequent exacerbation of inflammation, muscle breakdown, and excess fat production [120,121]. These changes further suppress the immune system, placing patients at increased risk of life-threatening infection and sepsis [120,121]. Glucose oxidation can increase from 4–5 to 7 g/kg/d after thermal injury [[121], [122], [123]] with nearly all burn patients exhibiting some degree of insulin resistance [124]. Administering insulin can improve lean body mass, bone mineral density, donor site healing, and decrease overall LOS in patients with severe burned injuries, although careful monitoring is required to avoid hypoglycemia [122]. In regard to fat composition, low-fat diets are thought to help avoid exaggerated immunosuppression, with many experts speculating that increasing the proportion of ω-3 fatty acids enhances immunity, reduces hyperglycemic episodes, and decreases ICU stays [121,122]. However, a recent systematic review and meta-analysis on this topic was unable to appreciate the benefit of ω-3 support in patients with burns [125]. Further studies are needed to validate whether altering lipid composition confers any clinical benefit. Protein catabolism is a hallmark of burn injuries, with patients oxidizing amino acids at a rate 50% higher than baseline [121]. Supplementing protein on the order of 1.5–2 g/kg/d increases protein synthesis and reduces negative nitrogen balance [121,122]. Protein rates exceeding this have not demonstrated any additional benefits [121,122].

Glutamine supplementation has been suggested to decrease rates of infection, hospital LOS, and mortality in patients with severe burns. The 2018 ESPEN guidelines recommend additional enteral doses of glutamine in patients with burns involving >20% body surface area [20]. However, several of the studies guiding this recommendation have significant flaws in their design and outcomes [121]. A recent international, multicenter RCT found no reduction in time to discharge from the hospital and no differences in 6-mo mortality, hospital LOS, or incidence of bacteremia when compared with placebo [126]. As such, the role of glutamine supplementation in severe burn injuries is now being reexamined. The study demonstrated similar adverse event rates among those who did and did not receive glutamine, so administration is unlikely to cause harm in these patients [126].

Thermal injuries cause significant oxygen-free radical generation and endothelial injury, so vitamin and mineral supplementation is thought to potentially protect against oxidative stress and improve wound healing [127]. Vitamins A, C, and D and iron, copper, selenium, and zinc are known to improve wound healing and immune function, so their supplementation is of interest for patients with burn injuries [122]. Resuscitation with vitamin C–enriched fluids may decrease total fluid requirements, wound edema, and severity of respiratory dysfunction, although it does increase the risk of postburn acute kidney injury [36,128]. Supplementation of calcium, magnesium, and vitamins A, B-1, B-6, B-12, C, D, and E has been shown to lower the risk for wound infection (30% compared with 77.4%; *P* < 0.001), sepsis (13.3% compared with 41.9%; *P* = 0.021), and prolonged hospitalization (51.80 compared with 76.81 d; *P* = 0.025) when compared with nonsupplemented patients [129]. Nevertheless, no evidence-based practice guidelines currently exist for the supplementation of vitamins and minerals after thermal injuries, and further studies are indicated to evaluate a clinically meaningful benefit. Given the limited risk of low-dose mineral supplementation, they may be considered in patients with severe burns [128]. In major burn patients, particularly those with renal failure requiring continuous renal replacement therapy for >5 d, monitoring of key micronutrients (especially copper, selenium, vitamins B-1, B-6, and C, and carnitine) lost via the burn wound and via continuous renal replacement therapy is encouraged [105,130,131]. All key recommendations discussed in this section are highlighted in Table 2 [[5], [6], [7], [8], [9], [10], [11], [12],^16^,^20^,^21^,^36^,^40^,[61], [62], [63], [64], [65], [66],[72], [73], [74], [75], [76], [77], [78], [79], [80], [81], [82], [83], [84], [85], [86],^92^,^98^,^104^,^105^,^112^,[116], [117], [118],[120], [121], [122],^124^,^126^,[128], [129], [130], [131]].

**TABLE 2:** Key recommendations for specific patient populations.

Nutritional component	Recommendations
Oral nutrition vs EN vs PN	- Feeding of any type should be initiated immediately after surgery without delay [[8], [9], [10], [11], [12],^16^] - Oral nutrition is always preferred when feasible - EN and PN may be used interchangeably without any increased risk of infection with PN [[72], [73], [74], [75], [76]] - Burn patients: EN within 24 h [118,120,121] - Open abdomens: early EN or PN recommended [[72], [73], [74], [75], [76], [77],^117^] - CRS/HIPEC: immediate PN until oral intake can cover daily requirements [92]
Glucose	- Administration of carbohydrate-containing clear liquids ≤2 h before surgery [[5], [6], [7],[61], [62], [63], [64], [65], [66]] - Formulate using maltodextrin to reduce solution osmolality - Burn patients: administration of insulin can improve lean body mass, bone mineral density, donor sit healing, and decrease LOS, though must be carefully monitored for hypoglycemia [122,124]
Protein	- Critically ill patients: 1.2–1.5 g/kg protein equivalents/d or lower [20,21,40,98,104] - CRS/HIPEC: 1.2–2 g/kg protein equivalents/d [92] - Burn patients: 1.5–2 g/kg protein equivalents/d [120,121] - Surgically open abdomens: ≤2.5 g/kg protein equivalent/d [112,116]
Fat	- Potential to enhance immunity, reduce hyperglycemia, reduce ICU LOS, particularly in burn patients, although more research is needed to evaluate impact [121,122]
Glutamine	- Potential to decrease hospital LOS, infections, and mortality, particularly in burn patients [20,[78], [79], [80],^121^,^126^] - More research is needed to evaluate impact, although there is little harm in administration [126]
Arginine	- Found to reduce infectious complications and hospital LOS when given at any point in the peri-operative period [[81], [82], [83], [84], [85], [86]] - Formally recommended by ESPEN for cancer patients [16,85,86]
Vitamin and Minerals	- Vitamins A, B-1, B-6, B-12, C, D, and E as well as iron, copper, selenium, zinc, and magnesium are thought to improve wound healing and immune function [36,122,128,129] - More research is needed to evaluate impact, although there is little harm in low-dose administration [128] - Burn patients: monitoring of key nutrients in major burn patients, particularly those on CRRT [105,130,131]

Abbreviations: CRRT, continuous renal replacement therapy; CRS, cytoreductive surgery; EN, enteral nutrition; HIPEC, hyperthermic intraperitoneal chemotherapy; ICU, intensive care unit; LOS, length of stay; PN, parenteral nutrition.

## Conclusion

The data regarding the clinical benefit of nutritional optimization is clear, so identifying high-risk patients and intervening in the perioperative period is of utmost importance in providing comprehensive surgical care. It is important for surgeons to understand the applicable prognostic tools (Table 1) and considerations to make when providing recommendations based on their patient population (Table 2). Collaboration with a multidisciplinary team for personalized dietary plans and advanced protocols for specific operations and patient groups will allow patients to reap the maximal benefits of perioperative nutrition optimization. Given the low risk, high reward nature of perioperative nutritional intervention and the large sector of patients who fail to be identified as malnourished during the operative period, the development of dietitian-driven clinical follow-up for patients to standardize nutritional optimization across the spectrum of surgical care may be a beneficial step for future care. A model of this was developed at Duke University, called the Perioperative Enhancement Team Nutrition Clinic, where multidisciplinary collaboration allowed for a structured pathway for malnutrition assessment and treatment driven by registered dietitians seeks to enhance surgical care [132]. These platforms may also serve as a launchpad for future research on specific foods and interventions for the various categories of surgical patients.

## Author contributions

The authors’ responsibilities were as follows—OH: designed the research, wrote the paper, and hold primary responsibility for final content; NA, AT: designed the research and wrote the paper; AR: wrote the paper; BS, NTN: edited the paper; PEW: wrote and edited the paper; and all authors: have reviewed and approve this manuscript submission.

## Funding

The authors reported no funding received for this study.

## Conflict of interest

PEW reports receiving investigator-initiated grant funding or unrestricted educational grants related to this work from the National Institutes of Health, Department of Defense, Abbott, Baxter, and Fresenius; has served as a consultant to Abbott, Fresenius, Baxter, Mend, and Nutricia for research related to this work; received unrestricted gift donations for nutrition research from Musclesound and DSM; and has received honoraria or travel expenses for CME lectures on improving nutrition care from Abbott, Baxter, Fresenius, Danone-Nutricia, DSM, and Nestle. The other authors report no competing interests.
